# Autocrine Signaling of NRP1 Ligand Galectin-1 Elicits Resistance to BRAF-Targeted Therapy in Melanoma Cells

**DOI:** 10.3390/cancers12082218

**Published:** 2020-08-08

**Authors:** Sabrina Rizzolio, Simona Corso, Silvia Giordano, Luca Tamagnone

**Affiliations:** 1 Candiolo Cancer Institute-FPO, IRCCS, 10060 Candiolo, Italy; sabrina.rizzolio@ircc.it (S.R.); simona.corso@ircc.it (S.C.); silvia.giordano@ircc.it (S.G.); 2 Department of Oncology, University of Torino Medical School, 10060 Candiolo, Italy; 3 Department of Life Sciences and Public Health, Università Cattolica del Sacro Cuore, 00168 Rome, Italy; 4 Fondazione Policlinico Universitario “A. Gemelli”, IRCCS, 00168 Rome, Italy

**Keywords:** galectin, neuropilin, melanoma, cancer therapy, molecular biology, cell signaling, oncogenic signaling, autocrine signaling, EGFR, BRAF

## Abstract

Melanoma cells addicted to mutated BRAF oncogene activity can be targeted by specific kinase inhibitors until they develop resistance to therapy. We observed that the expression of Galectin-1 (Gal-1), a soluble ligand of Neuropilin-1 (NRP1), is upregulated in melanoma tumor samples and melanoma cells resistant to BRAF-targeted therapy. We then demonstrated that Gal-1 is a novel driver of resistance to BRAF inhibitors in melanoma and that its activity is linked to the concomitant upregulation of the NRP1 receptor observed in drug-resistant cells. Mechanistically, Gal-1 sustains increased expression of NRP1 and EGFR in drug-resistant melanoma cells. Moreover, consistent with its role as a NRP1 ligand, Gal-1 negatively controls p27 levels, a mechanism previously found to enable EGFR upregulation in cancer cells. Finally, the combined treatment with a Gal-1 inhibitor and a NRP1 blocking drug enabled resistant melanoma cell resensitization to BRAF-targeted therapy. In summary, we found that the activation of Galectin-1/NRP1 autocrine signaling is a new mechanism conferring independence from BRAF kinase activity to oncogene-addicted melanoma cells.

## 1. Introduction

Melanoma cells carrying the mutated BRAF oncogene become addicted to its constitutive activity, which can be therapeutically targeted by kinase inhibitors, such as vemurafenib or its analog PLX-4720, achieving cytostatic and cytotoxic effects [1,2]. However, upon prolonged treatment with these drugs, melanoma cells almost inevitably develop adaptive drug resistance through the activation of alternative pathways, sustaining cell viability [3,4,5,6]. In particular, we have previously demonstrated that melanoma cells treated with BRAF inhibitors upregulate the cell surface receptor Neuropilin-1 (NRP1), which elicits an EGFR-dependent mechanism of adaptive resistance to therapy [7]. Additional studies by our group and by others have further demonstrated the broad relevance of NRP1 in promoting signaling cascades that support cancer cell growth and resistance to therapies [8,9,10,11].

NRP1 is a multifunctional receptor interacting with a range of extracellular ligands [12], which might represent relevant triggers of NRP1-dependent tumor malignancy and molecular targets for restoring drug sensitivity. In this respect, VEGF-dependent neuropilin signaling has been reported to play a relevant role in cancer; however, we have previously shown that VEGF is unlikely to be implicated in establishing NRP1-dependent melanoma resistance to BRAF inhibitors [7].

In the present study, we have, thus, investigated if other extracellular signals might trigger NRP1-dependent tumor malignancy, and found that a relatively poorly studied NRP1-ligand, Galectin-1 (Gal-1) [13,14], is upregulated in melanoma cells upon the onset of resistance to BRAF-targeted drugs. Gal-1 belongs to a family of secreted ligands that are known to regulate diverse biological processes in cancer [15,16], but which have not been previously implicated in the responsiveness to oncogene-targeted therapies. Here, we found that Gal-1 is largely responsible for NRP1-driven melanoma resistance to therapy and that its targeting with a small molecule inhibitor complemented the effect of NRP1-blockers aimed at resuming drug sensitivity.

## 2. Results

### 2.1. The Soluble NRP1-Ligand Galectin-1 Is Upregulated in Melanoma Cells Resistant to Targeted Therapy

**Figure 1:**
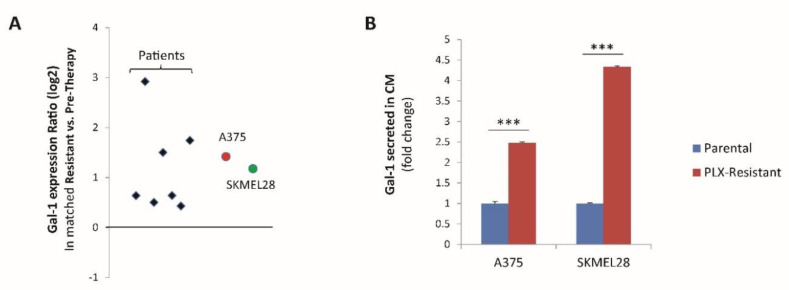
Galectin-1 upregulation in melanoma samples and cell lines resistant to BRAF-targeted therapy. (**A**) The graph shows Galectin-1 (LGALS1 gene) mRNA expression variation in paired melanoma samples (obtained from seven patients, indicated by black diamonds) before treatment with BRAF inhibitors and after the onset of secondary resistance (Log2 ratio resistant/pretherapy), as well as in A375 and SK-MEL-28 melanoma cells before and after developing resistance to 2μM PLX-4720 (Log2 ratio Resistant/Parental; *n* = 3). (**B**) Analysis of Galectin-1 protein levels (by PikoKine ELISA assay) in conditioned media (CM) derived from either parental or PLX4720-resistant A375 and SK-MEL-28 (PLX-resistant) cells. Data are normalized to levels detected in the respective parental cells and represent the average of a technical duplicate. Statistical significance was assessed by *t*-test, comparing parental and their derived PLX-resistant cell lines; *** *p* < 0.0001.

We have previously shown that melanoma cells resistant to BRAF-targeted therapy acquire NRP1 neo-expression, driving an intrinsic mechanism of drug refractoriness [7]. However, the potential role of extracellular ligands eliciting NRP1 activity in drug-resistant melanoma cells was not elucidated. Actually, preliminary data suggested that the conditioned medium of PLX4720-resistant melanoma cells contained bona fide soluble factors capable of promoting resistance to BRAF inhibitors (Figure S1A). However, in our previous study, we reported that this NRP1-driven drug resistance was not dependent on VEGF-A [7], which is a major autocrine NRP-1 ligand implicated in sustaining viability and self-renewal of cancer cells. Therefore, we aimed to identify the alternative autocrine ligands potentially triggering NRP1-dependent resistance to BRAF-targeted therapy in melanoma. Our attention was caught by the relatively poorly investigated NRP1 ligand Galectin-1 (Gal-1), encoded by the gene *LGALS1* [14,17], which has been associated with cancer progression in numerous studies and is also considered a potential therapeutic target [18,19]. Interestingly, we found a consistent upregulation of Galectin-1 mRNA levels in a set of paired melanoma samples obtained from the same patients before BRAF-targeted therapy and upon the onset of drug resistance (Figure 1A and Figure S1B); importantly, NRP1 expression also increased in most resistant tumors (Figure S1B), which was consistent with previous findings [7]. In full concordance, Galectin-1 expression was also significantly increased in A375 and SK-MEL-28 melanoma cells that developed resistance in culture upon prolonged treatment with escalating concentrations of the BRAF inhibitor PLX-4720 (Figure S1C and Figure 1A), thus recapitulating the process occurring in patients. This finding was further confirmed by the significant increase of Gal-1 secretion in the conditioned medium of PLX-4720-resistant (PLX-resistant) vs. parental cancer cells (Figure 1B). Intriguingly, this adaptive upregulation of Gal-1 expression in drug-resistant melanoma cells paralleled that reported for its receptor NRP1 [7] (see Figure S1D). Together these data highlight Gal-1 as a potential mediator of NRP1 autocrine signaling in drug-resistant melanoma cells and tumors.

### 2.2. Galectin-1 Is a Novel Driver of Resistance to BRAF-Targeted Therapy in Melanoma

**Figure 2:**
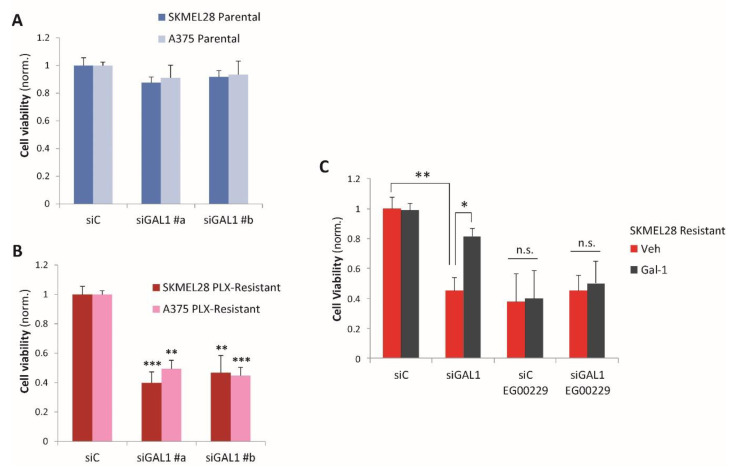
Increased Gal-1 expression sustains refractoriness to BRAF-targeted therapy. (**A**) Cell viability of parental A375 and SK-MEL-28 (by Cell Titer Glo Assay) upon Galectin-1 (LGALS1) knock-down with targeted siRNAs (siGAL1, two different targeting sequences: #a or #b) or treatment with control siRNAs (siC) (*n* = 3). (**B**) Cell viability of PLX4720-Resistant A375 and SK-MEL-28, upon Galectin-1 knock-down with targeted siRNAs as in panel A (*n* > 3), in the presence of PLX-4720 2 μM. Averaged values (±SD) were normalized to the respective conditions of the control siRNA treatment (siC), and statistical significance (versus respective controls for each cell line) was assessed by *t*-test; *** *p* < 0.0001; ** *p* < 0.005. (**C**) The viability of PLX4720-resistant SK-MEL-28, either Galectin-1-depleted (siGAL1) or control (siC), was assessed in the presence (Gal-1) or absence (Veh) of recombinant Gal-1 (1 μg/mL), with or without the NRP1 inhibitor EG00229 (12.5 μM), in the presence of PLX-4720 (2 μM). Viability was assayed after 72 h of treatment (*n* = 5). Statistical significance was assessed by *t*-test; * *p* < 0.01; ** *p* < 0.005.

In order to assess whether Gal-1 upregulation is functionally relevant in melanoma cells, we knocked down its expression by RNA interference in both parental and PLX-resistant A375 and SK-MEL-28 (validated by qPCR analysis, see Figure S2A,B). We observed that Gal-1 is basally expressed in parental drug-sensitive melanoma cells, but its silencing had no significant impact on their viability in the absence of BRAF inhibitors (Figure 2A), suggesting that in this condition Gal-1 autocrine signaling is either disabled or irrelevant for growth. In contrast, drug-resistant melanoma cells subjected to Gal-1 knock-down maintained sensitivity to the BRAF inhibitor PLX-4720 and their viability decreased remarkably (Figure 2B). Interestingly, drug-resistant SK-MEL-28 (releasing around 10-fold higher Gal-1 levels compared to A375) revealed strict dependence on Gal-1 expression, even though only partial gene knock-down could be achieved in these cells (Figure S2A,B). Cell viability loss caused by Gal-1 silencing in melanoma cells resistant to therapy was rescued in the presence of the recombinant purified Gal-1 added to the culture medium, indicating the specificity of the observed effects (Figure 2C, siGAL1 black bar). These data suggest that in melanoma cells resistant to PLX-4720, autocrine Gal-1 signaling is critical to support viability in the presence of the drug, featuring a previously unknown mechanism of refractoriness to BRAF-targeted drugs in melanoma.

We have previously shown that drug resistance in these cells is dependent on NRP1 and may be hindered by treatment with the NRP1-inhibitor EG00229 [7]. Intriguingly, Gal-1 knock-down did not have an additive effect in PLX-resistant cells treated with EG00229 (Figure 2C, siGAL1-EG00229 red bar) and recombinant Gal-1 could not rescue cell viability upon concomitant NRP1 blockade (Figure 2C, siGAL1-EG00229 black bar); these data indicate a mechanistic role of the NRP1 receptor in mediating Gal-1 autocrine signaling in drug-resistant melanoma cells. Conversely, parental drug-responsive cells, which have barely detectable levels of NRP1, were not dependent on Gal-1 expression (Figure 2A); moreover, the addition of recombinant Gal-1 could not obstruct the cytostatic activity of PLX-4720 in these cells (Figure S2C,D). Altogether, our data strongly suggest that Gal-1-induced resistance to therapy in melanoma cells is dependent on concomitant NRP1 receptor upregulation.

### 2.3. Galectin-1 Promotes NRP1 and EGFR Upregulation in Drug-Resistant Melanoma Cells

**Figure 3:**
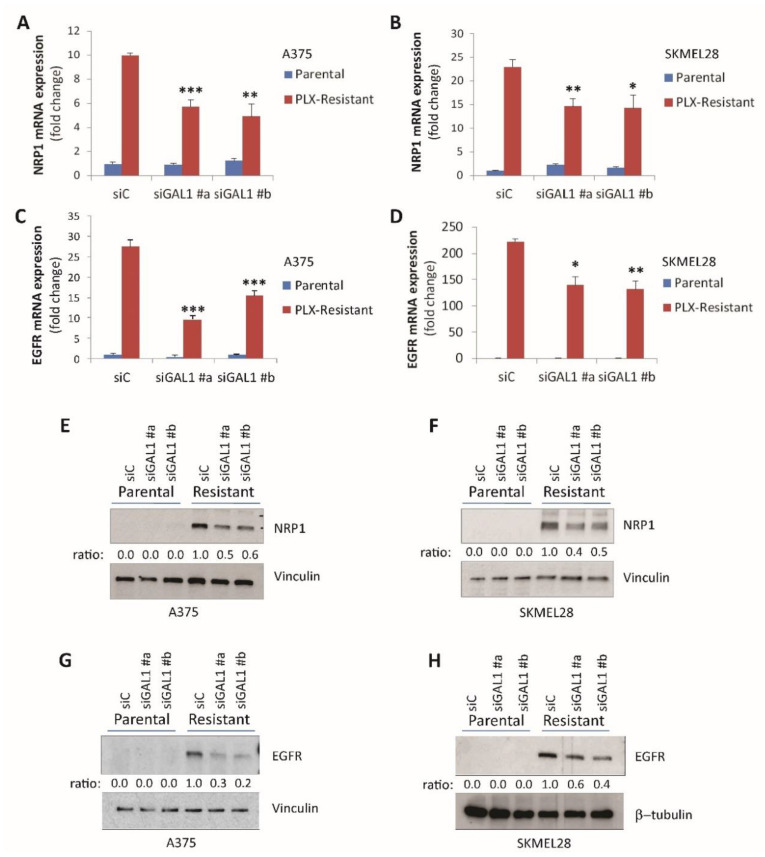
Gal-1 silencing downregulates NRP1 and EGFR expression. (**A**,**B**) The qPCR analysis of NRP1 expression in parental and PLX-4720-resistant A375 cells (A) and SK-MEL-28 (B) cells subjected to Gal-1 silencing by two different siRNAs (as in Figure 2; averaged values (±SD) were normalized to respective siC-transfected cells). Statistical significance was assessed by *t*-test by comparison with respective siC-treated conditions: * *p* < 0.01; ** *p* < 0.005; *** *p* < 0.0001. (**C**,**D**) The qPCR analysis of EGFR expression in the same parental and PLX-4720-resistant A375 and SK-MEL-28 cells subjected to GAL1 silencing analyzed in the previous panels (values normalized to respective siC-transfected cells). Statistical significance was assessed by *t*-test by comparison with respective siC-treated conditions: * *p* < 0.01; ** *p* < 0.005; *** *p* < 0.0001. (**E**,**F**) NRP1 expression was assessed by immunoblotting in the same cells subjected to GAL1 silencing analyzed in the previous panels; vinculin provided protein loading controls. The band intensity ratio was calculated and normalized to siC-resistant cells (results from an experiment representative of at least three repetitions). (**G**,**H**) EGFR expression was assessed by immunoblotting in the same cells analyzed above; vinculin or β-tubulin provided protein loading controls. The band intensity ratio was calculated and normalized to siC-resistant cells (results from an experiment are representative of at least three repetitions).

Since both Gal-1 and its receptor NRP1 were upregulated in melanoma cells upon drug resistance onset, we asked whether their relative expression was linked in a signaling circuit. In fact, NRP1 expression was basally very low in parental BRAF-addicted melanoma cells A375 and SK-MEL-28, and Gal-1 silencing did not significantly impact NRP1 levels (Figure 3A,B). In contrast, Gal-1 silencing significantly curbed that NRP1 upregulation observed in PLX-resistant melanoma cells at both the mRNA (Figure 3A,B) and protein levels (Figure 3E,F), thus suggesting a positive loop mediated by autocrine ligand production.

**Figure 4:**
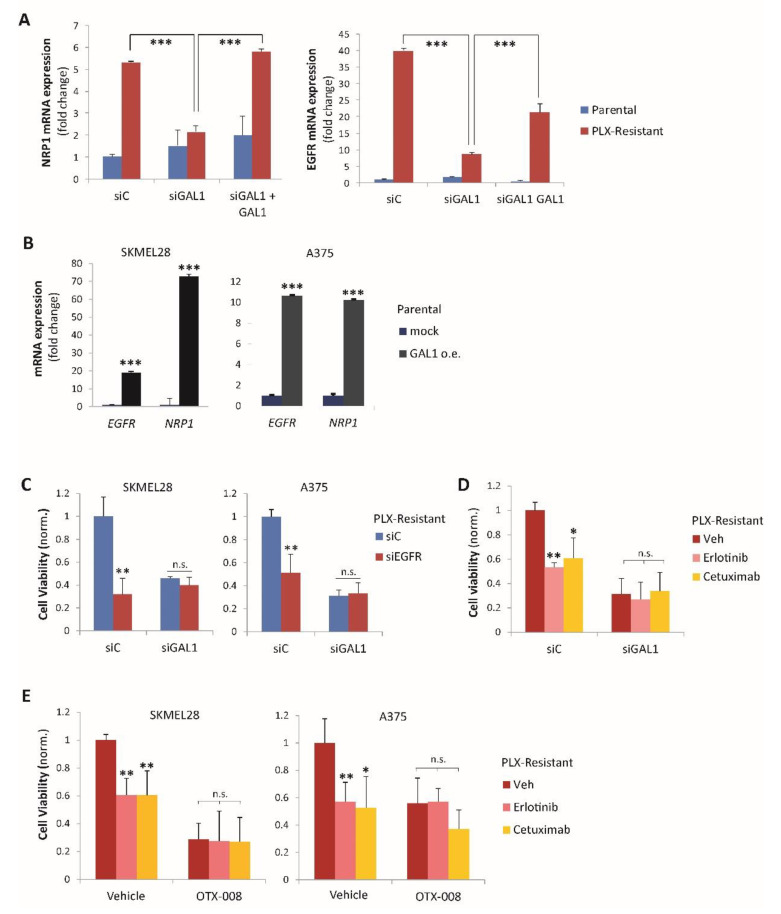
Galectin-1 promotes NRP1 and EGFR expression. (**A**) The qPCR analysis of *NRP1* (left) and *EGFR* (right) mRNA levels in parental or PLX-4720-resistant A375 cells, either control or subjected to Galectin-1 silencing, in the presence or absence of recombinant Gal-1 at a concentration of 1 μg/mL (*n* = 3). The statistical significance was assessed by *t*-test; *** *p* < 0.0005. Figure S3A shows consistent results obtained in the SK-MEL-28 cell model. (**B**) The qPCR analysis of *NRP1* and *EGFR* mRNA levels in SK-MEL-28 and A375 parental cells transfected with cDNA encoding Galectin-1 (GAL1 overexpressing, o.e.) or mock. The statistical significance was assessed by *t*-test by comparing the expression of the indicated genes in Gal-1 transfected vs. control cells; *** *p* < 0.0001. (**C**) Viability of SK-MEL-28 and A375 melanoma cells resistant to PLX-4720 (and maintained in the presence of the drug), subjected to Gal-1 knock-down (siGAL1) or control (siC), and further subjected (or not) to EGFR targeting with siRNA (siEGFR; achieving 0.3-fold average knock-down verified by qPCR). The statistical significance was assessed by *t*-test by comparing siEGFR-treated condition with the respective control conditions; ** *p* < 0.005. (**D**) The viability of A375 melanoma cells resistant to PLX-4720 (in the presence of the drug), either subjected to Gal-1 knock-down (siGAL1) or control (siC), was assessed upon co-treatment with the indicated EGFR inhibitors (erlotinib 100 nM or cetuximab 1 µg/mL) or with the vehicle alone. The statistical significance was assessed by *t*-test by comparing the EGFR-inhibitor treated with the respective untreated cells; ** *p* < 0.001, * *p* < 0.02. (**E**) By analogy to the previous panel, the viability of SK-MEL-28 and A375 cells resistant to PLX-4720 in the presence or absence of the Gal-1 blocking drug OTX-008 was assessed upon co-treatment with the indicated EGFR inhibitors or with the vehicle alone. The statistical significance was assessed by *t*-test by comparing cells exposed to EGFR-inhibitors with the respective controls; ** *p* < 0.005, * *p* < 0.05.

NRP1 was previously shown to mediate EGFR upregulation, driving resistance to targeted therapies, such as those inhibiting BRAF in melanoma cells [7]. We, thus, asked whether Gal-1 was also involved in controlling EGFR expression in these cells. Indeed, Gal-1 silencing significantly curbed the EGFR upregulation observed in PLX-resistant cells (Figure 3C,D,G,H). Notably, the gene regulatory effect driven by Gal-1 knock-down could be rescued by the addition of exogenous recombinant Gal-1 (Figure 4A and Figure S3A). In complementary experiments, both A375 and SK-MEL-28 parental melanoma cells transfected to express exogenous Gal-1 also attained consistent NRP1 and EGFR upregulation (Figure 4B).

The mechanistic relevance of Gal-1-driven EGFR upregulation in drug-resistant cells was demonstrated by the impact on cell viability of either EGFR-targeted siRNAs or catalytic inhibitors (Figure 4C,D and Figure S3B). Notably, such adaptive dependence on EGFR activity was prevented by Gal-1 knock-down (Figure 4C,D), confirming its role as an upstream regulator of the pathway. Analogous results were obtained upon treatment with the validated Gal-1 peptidomimetic inhibitor OTX-008 (Figure 4E) [20]. Not surprisingly, as for Gal-1 silencing, EGFR impairment did not basally affect the viability of drug-sensitive parental melanoma cells (Figure S3B).

It was previously reported that NRP1 negatively controls the expression of p27/Kip1 cyclin-dependent kinase inhibitor in cancer cells, a mechanism required to unleash the pathway leading to EGFR upregulation in PLX-resistant melanoma cells [7,21]. Interestingly, while p27 expression is suppressed in drug-resistant cells, Gal-1 silencing rescued p27 mRNA and protein levels to match those found in parental cells (Figure S4A,B); this upregulation is similar to that reported upon NRP1 knock-down [7], and is further consistent with the role of Gal-1 as a NRP1-activator in this context. The mechanistic relevance of p27 suppression by Gal-1 signaling in drug-resistant cells was demonstrated by the evidence that p27 knock-down by siRNAs could rescue the impairment of drug resistance caused by Gal-1 silencing (Figure S4C). Further experiments indicated that the downregulation of p27 is accountable for Gal-1-driven EGFR induction in drug-resistant melanoma cells (Figure S4D).

### 2.4. Gal-1 and NRP1 Inhibitory Drugs Cooperate to Promote Melanoma Cell Resensitization to BRAF-Targeted Therapy

**Figure 5:**
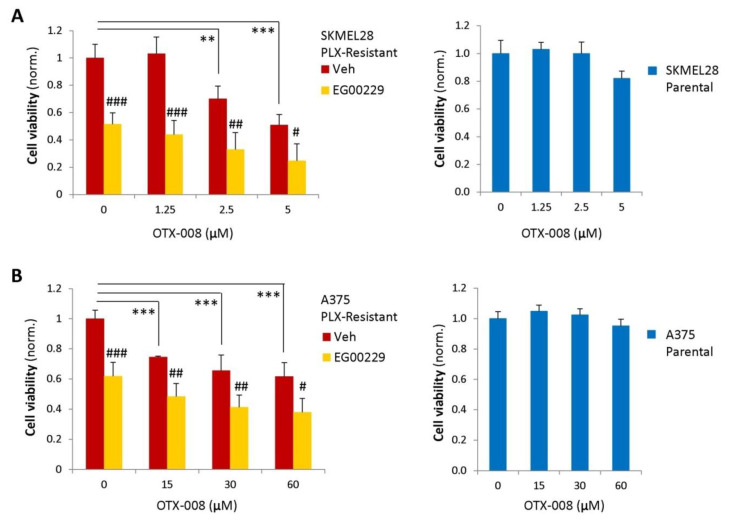
Gal-1 inhibition restores the efficacy of BRAF-targeted therapy in drug-resistant melanoma cells. (**A**) On the left is shown the viability of PLX4720-resistant SK-MEL-28 melanoma cells under treatment with BRAF-targeted drugs and exposed to increasing concentrations of OTX-008 (Gal-1 inhibitor), either in the absence or presence of 12.5 μM of EG00229 (*n* > 4). On the right is shown the impact of the same concentration range of OTX-008 on SK-MEL-28 parental cells. Averaged values (±SD) were normalized to respective siC-transfected cells. Statistical significance was assessed by *t*-test: ** *p* < 0.005; *** *p* < 0.0001. Moreover, *t*-test was used to compare EG00229-treated vs. respective vehicle-treated cells: # *p* < 0.01; ## *p* < 0.005; ### *p* < 0.0001. (**B**) The same analysis as above was carried out in PLX4720-resistant (left) and parental (right graph) A375 cells. Statistical significance was assessed by *t*-test, as in the previous panel: *** *p* and ### *p* < 0.0001; ## *p* < 0.005; # *p* < 0.01.

Although the peptidomimetic NRP1 inhibitor EG00229 could rescue responsiveness to BRAF-targeted therapy in resistant melanoma cells, drug resensitization was never complete [7]. We, therefore, aimed to target autocrine Gal-1 signaling in BRAF-inhibitor-resistant melanoma cells from a therapeutic perspective. Interestingly, the Gal-1 blocking drug OTX-008 elicited a significant resensitization of resistant cells to BRAF-targeted therapy (Figure 5A,B, red bars in left graphs). As observed for Gal-1 gene silencing, Gal-1 inhibitor treatment had no impact on parental PLX-4720-sensitive cells (Figure 5A,B, blue bars in right graphs); this is consistent with the conclusion that Gal-1 autocrine signaling is specifically relevant for mediating drug resistance in NRP1-expressing cells. Notably, OTX-008 cooperated with EG00229 to achieve resensitization to therapy of BRAF-inhibitor-resistant melanoma cells (Figure 5A,B, yellow bars in left graphs), comparable to the responses observed in parental drug-sensitive cells (check Figure S1C). The treatment with Gal-1 or NRP1 inhibitors, independently or in combination, did not induce cell toxicity per se in the absence of BRAF inhibitors, indicating that the observed therapeutic benefit was not due to non-specific off-target effects (Figure S5). Since multidrug therapeutic regimens have shown a reduced risk of non-specific adverse effects, the strong efficacy of NRP1/Gal-1 combined targeting offers a promising perspective for regaining therapeutic efficacy for BRAF inhibitors in melanomas that have developed drug-resistance.

## 3. Discussion

The onset of acquired resistance to targeted therapies is a challenge in clinical oncology. Drug resistance is accounted by several mechanisms, often involving the adaptive upregulation of alternative signaling pathways that sustain cancer cell viability [22,23]. We have previously reported that NRP1 can orchestrate such mechanisms in different tumor cell types. In particular, NRP1 elicits a novel signaling cascade that is primed by the downregulation of the p27/Kip1 cyclin-dependent kinase inhibitor and which leads to EGFR upregulation at the transcriptional level [7]. EGFR kinase activity was actually found to mediate adaptive drug resistance in melanoma cells [6,7]. Moreover, we reported that NRP1 expression is markedly upregulated in BRAF-inhibitor-resistant melanoma cells [7]; however, the mechanism triggering NRP1 activity was not elucidated.

Since NRP1 acts as a relatively promiscuous co-receptor molecule on the cell surface, we thought that this signaling cascade might be activated by a soluble ligand, potentially acting in an autocrine manner. By analyzing an accessible small series of paired melanoma samples, we found that a relatively poorly studied NRP1-ligand, Galectin-1, is induced upon the onset of resistance to BRAF inhibitors. This was confirmed in two distinct melanoma cell lines, where we found that Galectin-1 was driving the refractoriness to BRAF-targeted therapy. Mechanistically, this implied the adaptive upregulation of Gal-1 expression, concomitant with that of its receptor NRP1. In fact, Gal-1 knock-down in drug-resistant cells impaired the entire pathway—NRP1 and EGFR upregulation was blunted and p27 expression was restored. Conversely, Gal-1 overexpression could trigger this signaling circuit, promoting the upregulation of NRP1 and that of its downstream target EGFR in parental melanoma cells. The relevance of EGFR activity in this adaptive mechanism of resistance to therapy has been previously demonstrated [7]; notably, here we confirm that Gal-1 silencing prevents drug-resistant melanoma cells’ dependence on EGFR, consistent with its role as an initiator of this pathway (see Graphical Abstract).

Notably, parental melanoma cells sensitive to BRAF inhibitors were not affected by Gal-1-targeting siRNAs or a peptidomimetic inhibitor, which is consistent with the fact that the receptor NRP1 is almost undetectable and that the autocrine signaling circuit described here is not active in these cells. Such findings provide proof of the principle of specificity for this mechanism for drug-resistant melanoma cells, which could be exploited from a therapeutic perspective. Although our data indicate that Gal-1 and NRP1/EGFR upregulation is a frequent adaptive mechanism in melanoma cells treated with BRAF inhibitors, this signaling cascade may not be critical in every case of melanoma developing drug resistance. Thus, the assessment of NRP1 expression in tumor biopsies could be used as a surrogate indicator of its targetability in selected patients.

Galectins form a wide family of small secretable molecules, interacting with specific glycosylated proteins exposed on the cell surface or in the extracellular matrix [15]. They have been implicated in myriad biological processes relevant in cancer, including cell proliferation, cell adhesion, apoptosis, angiogenesis, and immunoregulation [16]. In particular, Gal-1 expression was positively associated with the malignant progression of diverse tumor types, including melanoma [24]; however, its relevance in conditioning the responsiveness to oncogene-targeted therapies was previously unknown. Thus, the activity promoting melanoma resistance to therapy established by this study further expands the importance of Gal-1 as a therapeutic target in cancer. In particular, we exploited the activity of the Gal-1 inhibitor OTX-008 as an anticancer agent. OTX-008 is an allosteric inhibitor that induces Gal-1 intracellular degradation [18], thereby strongly downregulating its extracellular release. Notably, OTX-008 has also been reported to interfere with NRP1-dependent signaling [25], consistent with its activity against Gal-1.

Gal-1 as a ligand for NRP1 was previously reported in the context of endothelial cells, where it was found to promote NRP1 association with VEGFR2 [14,26]. Since VEGFR2 is not expressed at significant levels in melanoma cells, we posited that NRP1 elicits a distinct signaling pathway upon Gal-1 engagement in this context. Actually, NRP1-ligand-binding domains are incompletely understood, and the region responsible for Gal-1 binding is currently unknown. For instance, while b1-b2 NRP1 domains have been implicated for the binding of both class 3 semaphorins and VEGF, mutational studies have demonstrated that distinct amino acid residues are selectively required to bind these ligands [27], consistent with the fact that they often mediate opposite functional activities. Notably, semaphorin-NRP1 signaling is not involved in sustaining cell viability, and we have previously shown that VEGF-NRP1 autocrine signaling is not implicated in the pathway leading to EGFR upregulation and drug resistance in melanoma cells [7]. In fact, NRP1 is well known to partner with a wide range of transmembrane receptor molecules [28], although the mechanisms gating its involvement in different complexes are poorly understood. While VEGF may stabilize the association of NRP1 with VEGF receptor kinases, and the semaphorins with their specific receptors called plexins, it is currently unknown which signaling molecules associate with NRP1 in response to Gal-1.

Gal-1 has been reported to interact with few glycosylated proteins exposed on the cell surface beyond NRP1. Importantly, NRP1 involvement in Gal-1-driven resistance to therapy in melanoma cells is indicated by different lines of evidence in our study. First, the knock-down of either Gal-1 or NRP1 showed the same functional impact on restoring drug sensitivity in melanoma cells; moreover, the effects of these treatments were not additive and were instead mutually exclusive, suggesting that the targeted effectors lie in the same signaling pathway. Second, drug resistance rescue in Gal-1-silenced cells by treatment with recombinant Gal-1 was dependent on NRP1. Third, Gal-1 knock-down did not impact the viability of drug-sensitive melanoma cells, which express negligible levels of NRP1.

Moreover, we have previously shown that the NRP1-dependent signaling cascade leading to EGFR transcriptional induction is primed by p27/Kip1 downregulation and prevented by forced p27 overexpression [7]. Besides, a negative regulation of p27 by NRP1 has been confirmed, sustaining cancer cell viability in different models [7,29,30]. Notably, here we confirmed that Gal-1 controls p27 levels in PLX-resistant melanoma cells, consistent with its role as an autocrine NRP1 activator.

The importance of NRP1 in regulating EGFR signaling in cancer cells has been described previously [7,28]. Here, we confirm that Gal-1 controls EGFR upregulation in drug-resistant melanoma cells via a NRP1-dependent mechanism. The effector role of EGFR kinase in this pathway is confirmed not only by gene knock-down experiments (Figure 4C), but also by combined treatments with Gal-1 and EGFR inhibitors (Figure 4D). In fact, both approaches increased the sensitivity to PLX-4720 in melanoma cells; the effects were not additive and rather mutually exclusive, suggesting that the molecules lie in the same signaling pathway. Notably, as with Gal-1 silencing, EGFR impairment did not impact the viability of drug-sensitive parental melanoma cells, which are independent of its kinase activity.

Considering the complexity of the galectin family, we cannot presently rule out the potential involvement of additional galectins beyond Gal-1 in melanoma resistance to therapy. Nonetheless, the converging results obtained by OTX-008 treatment and Gal-1 knockdown with siRNA lead to the solid conclusion that Gal-1 is pivotally implicated in melanoma cell resistance to BRAF-targeted therapy. Notably, OTX-008 activity is selective for Gal-1 versus Gal-3 (the other mostly studied family member), but potential interactions with other galectins have not been excluded at the present stage [31]. In fact, the combined effects of this drug with EG00229 may be compatible with additional NRP1-independent anticancer activities. Importantly, the observed efficacy of Gal-1 targeting in preclinical cancer models has stimulated its application for translational purposes [32,33]. Indeed, several Gal-1 inhibitors have been designed with potential clinical applications in cancer therapy [31,34]. In addition, recently both Gal-1 and NRP1 have been independently implicated in cancer immune escape and as putative targets for tumor immunotherapy [35,36,37], potentially featuring a pathway that deserves further investigation. Thus, Gal-1 autocrine signaling via NRP1 represents a druggable mechanism mediating melanoma cell resistance to BRAF inhibitors. These findings are a substantial bases for future studies, validating the efficacy of Gal-1/NRP1 targeting in vivo in preclinical models of melanoma.

## 4. Materials and Methods

### 4.1. Human Cell Lines

The A375 melanoma cell line was provided by American Type Culture Collection (ATCC). SK-MEL-28 melanoma cells were provided by NCI. All tested and authenticated cancer cell lines used in this study were cultured in the following media: RPMI for SK-MEL-28 and DMEM for A375 cells. The media were supplemented with 1% L-glutamine (2 mMol/L), 10% FBS (Sigma, St. Louis, MO, USA), penicillin (100 U/mL) and streptomycin (0.1 mg/mL)(Pen-Strep, cat. P0781, Sigma), then incubated in a humidified incubator with 5% CO_2_ at 37 °C. Cell lines were passaged in culture for less than 6 months after resuscitation.

### 4.2. Analysis of Galectin-1 mRNA Expression in Tumor Samples

We compared *LGALS1* mRNA levels in seven pairs of matched melanoma samples, each derived from the same patient pretreatment and after the onset of resistance to therapy with BRAF inhibitors. Four of these were described in the study published by Kwong and coworkers [38] (dataset accession: EGAD00001001306 at www.ebi.ac.uk), while another three were previously described [6].

### 4.3. Antibodies and Other Reagents

EGFR was detected with an antibody purchased from Enzo Life Sciences, Farmingdale, NY, USA (cat. ALX-804-064-C100). The p27/Kip1 antibody (ab32034) was purchased from Abcam, Cambridge, UK. Other antibodies applied in this study were anti-vinculin (V4505, Sigma) and anti-βtubulin (T4026, Sigma). Secondary antibodies were purchased from Promega (Madison, WI, USA) or Jackson Laboratories (Bar Harbor, ME, USA). PLX-4720 was purchased from Med Chem Express (Monmouth Junction, NJ, USA) and resuspended in DMSO as the vehicle. OTX-008 was purchased from Axon Medchem (Reston, VA, USA). Purified Galectin-1 (cat: 10290-HNAE) was purchased from Sino Biological Europe GmbH (Eschborn, Germany).

### 4.4. Establishment of Acquired Cancer Cell Resistance to Oncogene-Targeted Inhibitors

The protocol to establish A375 and SK-MEL-28 cell lines resistant to PLX-4720 has been previously described [7]. Briefly, we treated naïve (parental) melanoma cells with escalating concentrations of the drug (starting from 125 nM) until they acquired the ability to grow in the presence of 2 μM PLX-4720 at the same rate as parental cells in the absence of the drug.

### 4.5. Transient Cell Transfection with cDNA and siRNA

The cDNA- and siRNA-expressing constructs were transiently transfected in mammalian cells with Lipofectamine 2000 (for siRNAs) or Lipofectamine 3000 (for cDNAs) (Life Technologies, Carlsbad, CA, USA), according to the manufacturer’s instructions. LGALS1 expression plasmid (Galectin-1 cDNA ORF Clone, Human, C-His tag purchased from Sino Biological) was transfected in cells and positively selected with Hygromycin-B (H3274, Sigma). For “reverse cell transfection” experiments, the cells were transfected immediately after detachment. Briefly, the cells were detached with trypsin and counted. In the meantime, a mix of siRNA or cDNA, Opti-MEM medium, and Lipofectamine was prepared (according to the manufacturers’ instructions) and incubated at room temperature for 20 min. The transfection mix was dispensed first in a 96-well multiwell plate, immediately followed by the cell suspension (1–4 × 10^3^ per well, depending on cell line) in quadruplicate points. The transfected cells were directly analyzed in the wells (with cell viability assays) 72–96 h after transfection. The siRNA sequences applied are: siC (AAUUCUCCGAACGUGUCACGU); siGAL-1 #a (CCAGAUGGAUACGAAUUCA); siGAL-1 #b (GCUGCCAGAUGGAUACGAA). EGFR transcripts were targeted with a pool of two validated siRNA sequences (GCAGUGACUUUCUCAGCAA; GCAGUCUUAUCUAACUAUGAU). Similarly, the knock-down of p27 (*CDKN1B*) was achieved by a pool of silencer siRNAs (ID s2837, ID s2838) provided by Ambion Thermofisher (Waltham, MA, USA).

### 4.6. RNA Isolation and Real-Time PCR

Total RNA from tumor cell lines was isolated with the RNeasy Mini Kit (Qiagen), according to the manufacturer’s instructions. The cDNA preparation was done according to standard procedures, using M-MLV reverse transcriptase (Promega) and oligo-dT primers (Promega). Gene expression was measured using the following Taqman gene-specific probes from Thermofisher: *NRP1* (Hs00826128_m1), *EGFR* (Hs00193306_m1), and the housekeeping genes *GAPDH* (Hs04420632_g1) and β-actin (Hs99999903_m1). The expression of the following genes was assessed by means of SYBR-green specific primer pairs: p27/Kip1 (*CDKN1B* gene) (CTGAGGACACGCATTTGGT, GGGGAACCGTCTGAAACAT), Galectin-1 (*LGALS1* gene) (GACTCAATCATGGCTTGTGGTCTG, GCTGATTTCAGTCAAAGGCCACAC); and the housekeeping genes *GAPDH* (GAAGGTGAAGGTCGGAGTC, GAAGATGGTGATGGGATTTC) and β-actin (CACTCTTCCAGCCTTCCTTC, GTACAGGTCTTTGCGGATGT). Real-time PCR analysis was performed using the 7900HT Fast Real-time PCR System by Applied Biosystems (foster City, CA, USA).

### 4.7. Cell Viability Assays

Tumor cells were seeded in 96-well plastic culture plates at an initial density of 1–2 × 10^3^ cells per well (depending on the cell line) in the presence of the indicated drugs or vehicle (DMSO). Then, 72 h after cell seeding the medium was changed and cell viability was assessed by CellTiter-Glo Luminescent Cell Viability Assay (Promega), according to the manufacturer’s instructions. The number of viable cells was directly proportional to the luminescence signal recorded.

### 4.8. Conditioned Media Analysis by ELISA

Conditioned media were collected 72 h after cell seeding. Media were filtered to remove cell debris and stored at −80 °C (if needed). Galectin-1 concentrations in CM were measured by PikoKine ELISA assay (category EK0762; Boster Biological Technology, Pleasanton, CA, USA), according to the manufacturer’s protocol. Results were normalized on the total protein content in lysates of the corresponding producer cell dishes.

### 4.9. Western Blotting Analysis

Total protein extracts were obtained by cell lysis in LB-SDS buffer (25% Tris HCl 0.5 M pH 6.8, 25% SDS 10%, 50% ddH20). Protein concentrations were quantified by BCA Protein Assay kit (Thermofisher) and equal amounts of total cellular lysates were separated by SDS-PAGE and transferred to nitrocellulose membranes, which were blocked by incubation in 10% BSA. The membranes were incubated in the specific properly diluted primary antibody and then in the appropriate peroxidase-conjugated secondary antibody (Promega). Signal detection was done by enhanced chemioluminescence (ECL, Amersham Biosciences, GE Healthcare, Chicago, IL, USA). Band densitometry analysis was done with ImageJ software (NIH, Bethesda, MD, USA). All whole western blots are available in Figure S6.

### 4.10. Statistical Analysis

Experiments were repeated at least three times (biological replicates). Graphs show the average values of the experiments; error bars represent the standard deviation. Statistical significance was assessed using the two tailed Student’s *t*-test. Unless otherwise specified in the legend, asterisks indicate statistical significance according to the following legend: * *p* < 0.01; ** *p* < 0.001; *** *p* < 0.0001.

## 5. Conclusions

In summary, our work shows that Galectin-1 triggers an autocrine NRP1-dependent pathway in melanoma cells, driving adaptive refractoriness to BRAF inhibitors. The combined blockade of Galectin-1 and NRP1 in drug-resistant melanoma cells restored BRAF-addiction and sensitivity to therapy. Thus, Galectin-1/NRP1 signaling represents a druggable mechanism controlling adaptive resistance to therapy in melanoma cells. Future studies based on our findings will validate the efficacy of its targeting in vivo in preclinical models of melanoma.

## Supplementary Materials

The following are available online at https://www.mdpi.com/2072-6694/12/8/2218/s1, Figure S1: Viability of melanoma cells upon treatment with BRAF inhibitor, Figure S2: Gal-1 silencing in melanoma cells, Figure S3: NRP1 and EGFR regulation by Gal-1 and EGFR involvement in Gal-1 signaling, Figure S4: Gal-1-dependent regulation of p27 expression and its involvement in Gal-1 signaling, Figure S5: OTX-008 and EG00229 treatment of melanoma cells, Figure S6: Supplementary informations about Figure 3E,F and Figure S3B (uncropped immunoblots).
