# Supplementary material for: Autocrine Signaling of NRP1 Ligand Galectin-1 Elicits Resistance to BRAF-Targeted Therapy in Melanoma Cells
Source: Cancers (Basel). 2020 Aug 8;12(8):2218. doi: 10.3390/cancers12082218 (PMC7463444; doi:10.3390/cancers12082218)
Supplement: Supplementary file 1 [file cancers-12-02218-s001.pdf]

cancers

Supplementary Files

Autocrine Signaling of NRP1-Ligand Galectin-1 Elicits Resistance to BRAF-Targeted Therapy in Melanoma Cells

Sabrina Rizzolio, Simona Corso, Silvia Giordano and Luca Tamagnone

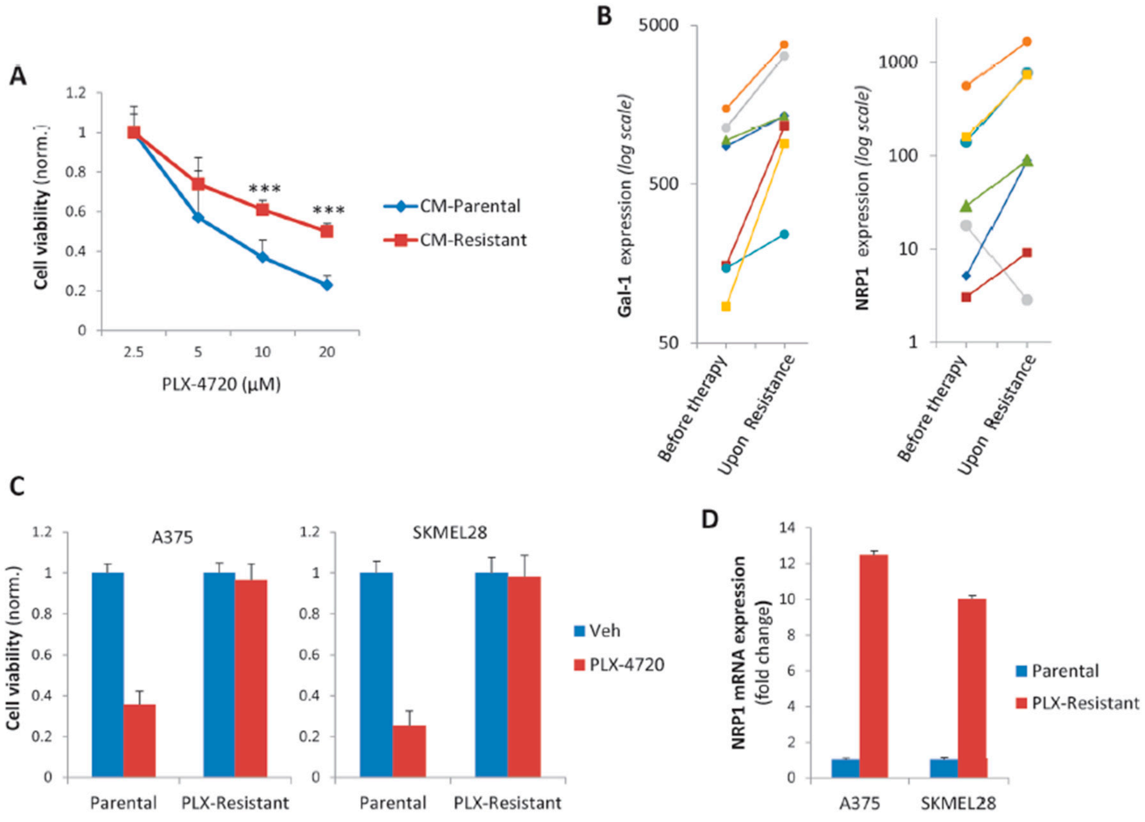

Figure S1. Viability of melanoma cells upon treatment with BRAF-inhibitor. (**A**) SK-MEL-28 melanoma cells refractory to treatment with 2 µM PLX-4720 were incubated for 72 h with increasing concentrations of the BRAF-inhibitor, in the presence of conditioned medium harvested from either parental drug-sensitive (CM-Parental) or therapy-resistant (CM-Resistant) cells. Cell viability was then assessed as described in Methods. (**B**) Expression of Gal-1 (graph on the left) and NRP1 (on the right) mRNA, in paired melanoma samples taken from the same patients before therapy with BRAF-inhibitors or after the onset of drug-resistance. On the Y axis (Log scale) are plotted RNA-Seq reads (as reported in public dataset). Color coded connectors and symbols indicate matched samples derived from the same patient. (**C**) The viability of A375 and SK-MEL-28 Parental (BRAF-inhibitor sensitive) and targeted therapy-Resistant cells was assessed (by Cell Titer Glo Viability Assay) in the presence (or absence) of 2 µM PLX-4720 BRAF inhibitor. (**D**) NRP1 expression was assessed by qPCR analysis in Parental and PLX-4720 Resistant A375 and SK-MEL-28 melanoma cells.

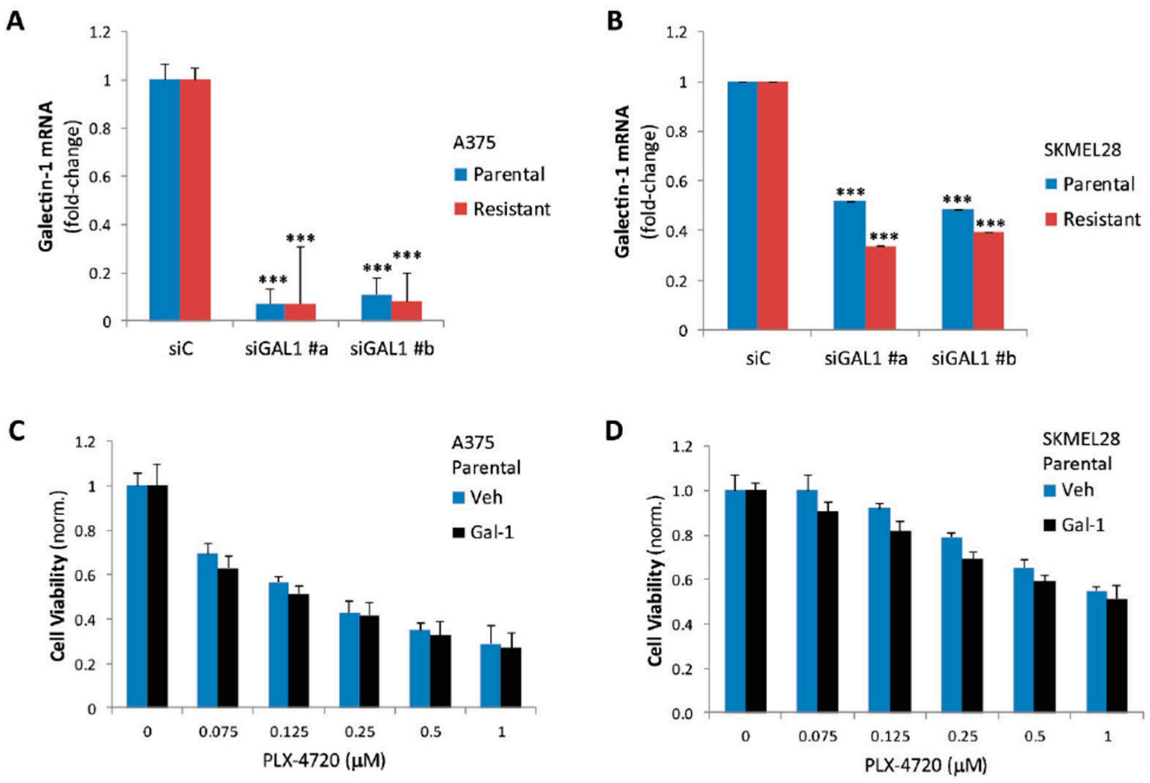

Figure S2. Gal-1 silencing in melanoma cells. (**A–B**) qPCR analysis of Galectin-1 expression in Parental and PLX-4720-Resistant A375 (**A**) and SK-MEL-28 (**B**) cells subjected to Galectin-1 knock-down (siGAL1) or treated with control siRNA (siC) (n = 4). Averaged values (± SD) values were normalized to respective siC-treated controls. The statistical significance was assessed comparing each series of siGAL1-treated samples with the respective siC control samples derived from the same cells, by Student's t-test: ***$p < 0.0001$. (**C–D**) Cell Viability of Parental A375 (**C**) and SK-MEL-28 (**D**) cells treated for 72 h with increasing concentration of the BRAF inhibitor drug PLX-4720, in presence or absence if recombinant Gal-1 (1 μg/mL).

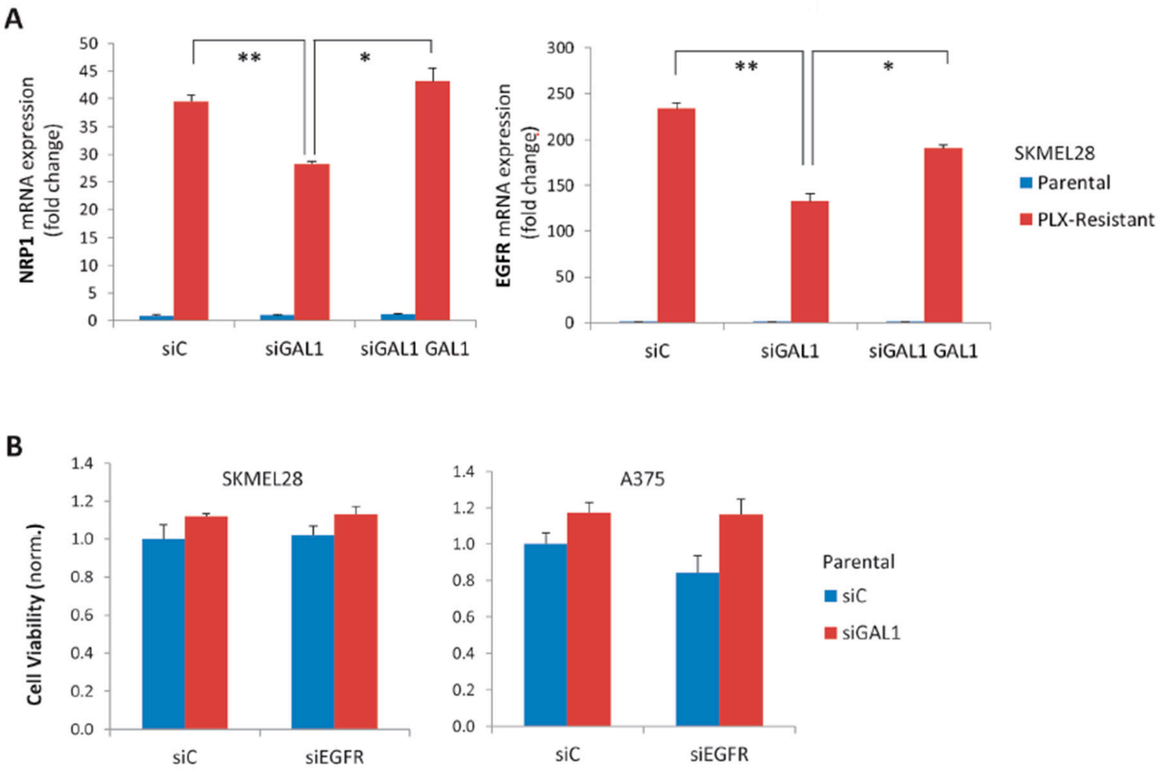

Figure S3. NRP1 and EGFR regulation by Gal-1 and EGFR involvement in Gal-1 signaling. (**A**) qPCR analysis of NRP1 (left) and EGFR (right) mRNA levels in Parental or PLX-4720-Resistant SK-MEL-28 cells, either control or subjected to Gal-1 silencing, in presence or absence of recombinant Gal-1 at a concentration of 1 µg/mL ($n = 3$). The statistical significance was assessed by *t*-test; ** $p < 0.005$, * $p < 0.05$. (**B**) The viability of Parental SK-MEL-28 and A375 melanoma cells was assessed upon Gal-1 knock-down with siRNAs, alone or in combination with a pool of two siRNAs targeting EGFR. Averaged values (± SD) were normalized to the respective conditions of control siRNA treatment (siC).

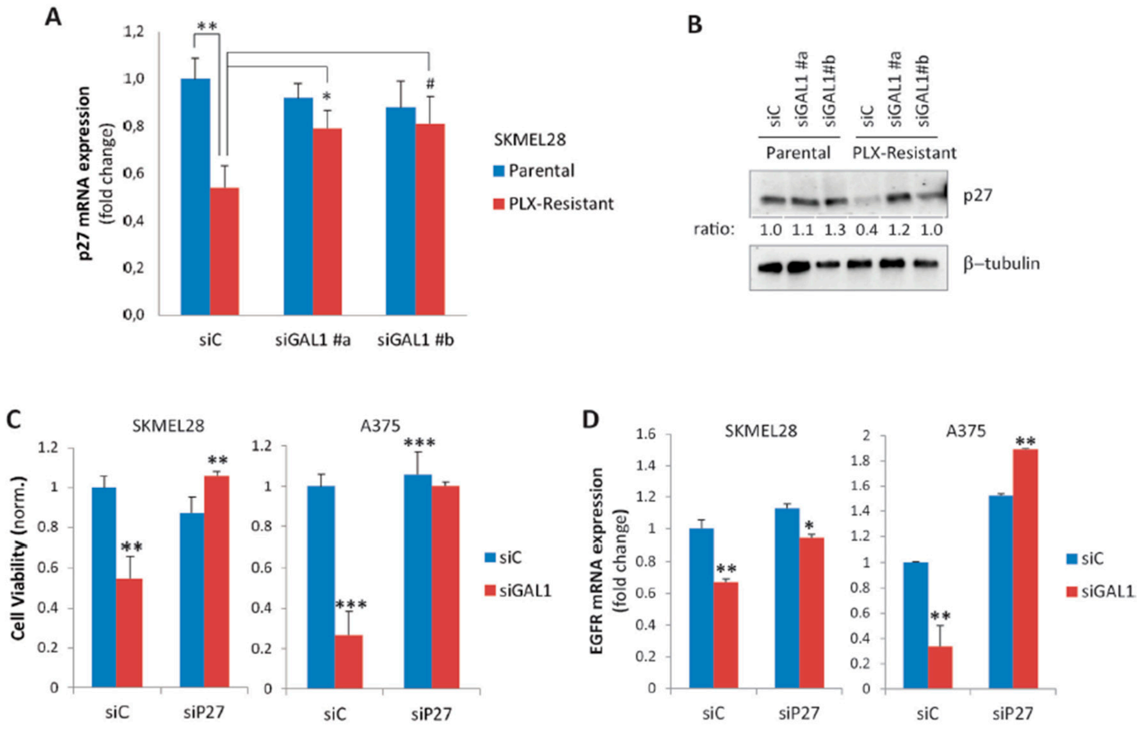

Figure S4. Gal-1-dependent regulation of p27 expression and its involvement in Gal-1 signaling. (**A**) qPCR analysis of p27 expression in SK-MEL-28 Parental or PLX-4720-Resistant cells, upon Gal-1 silencing (the same cells analyzed in main Fig. 2 and 3). Averaged values (± SD) were normalized to parental siC-treated controls ($n > 3$). Statistical significance was assessed by *t*-test: ** $p < 0.001$; * $p < 0.01$; # $p < 0.05$. (**B**) Western blotting analysis of p27 expression in SK-MEL-28 Parental or PLX-4720-Resistant cells, subjected to Gal-1 silencing (the same as in previous panel, and analyzed in main Fig. 2 and 3). β-tubulin provided a protein loading control, and band intensity ratio was calculated, normalized to first lane. Representative results of independent replica experiments. (**C**) The viability of PLX4720-Resistant SK-MEL-28 and A375 cells (maintained in the presence of the drug) was assessed upon Galectin-1 knock-down with targeted siRNAs alone or in combination with a pool of two siRNAs targeting p27 expression (achieving 0.1 fold average knock-down verified by qPCR). Averaged values (± SD) were normalized to the respective conditions of control siRNA treatment (siC), and statistical significance was assessed by *t*-test versus respective control conditions: *** $p < 0.0005$, ** $p < 0.005$. (**D**) EGFR expression levels were analyzed by qPCR in drug-Resistant SK-MEL-28 and A375 cells treated as described in panel C. The values were normalized to siC-transfected controls per each cell line, and the statistical significance was assessed by *t*-test comparing Gal-1 silenced cells with respective control conditions: ** $p < 0.005$, * $p < 0.01$.

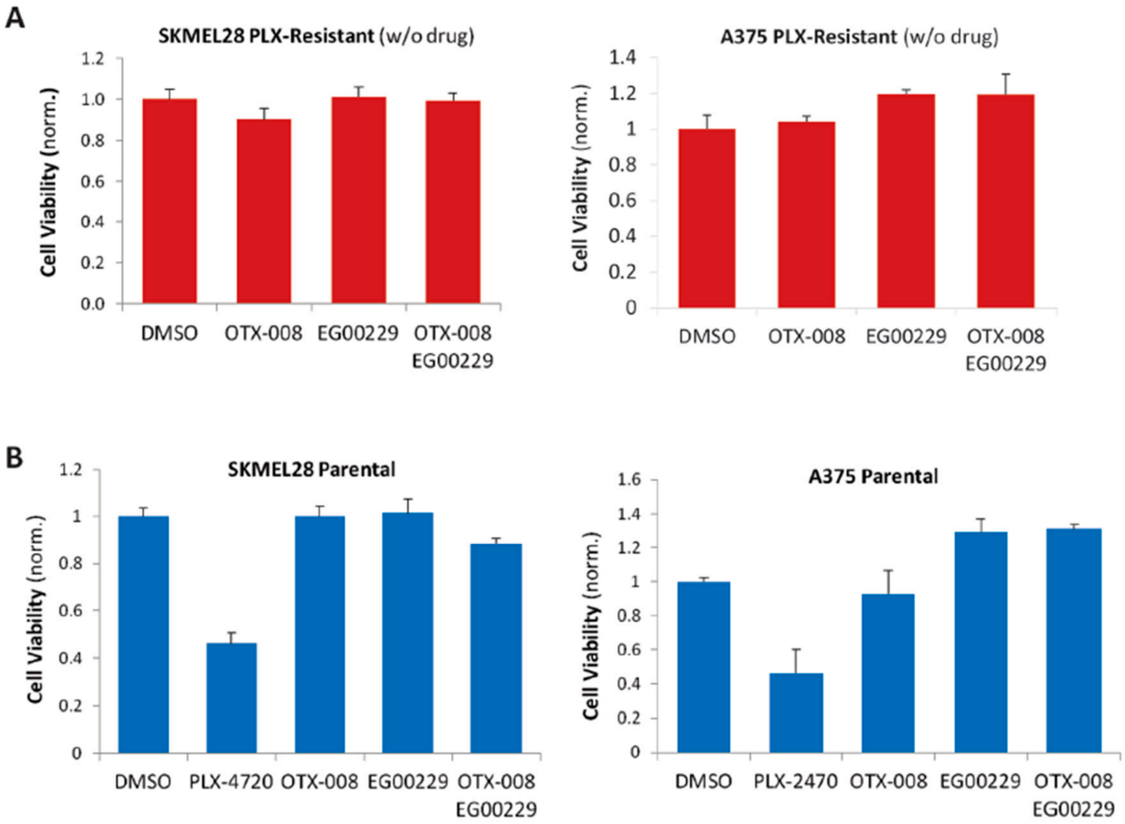

Figure S5. OTX-008 and EG00229 treatment of melanoma cells. (**A**–**B**) The viability of the indicated melanoma cells (SK-MEL-28 on the left and A375 on the right), either Resistant to PLX-4720 (but left in absence of the drug) (panel **A**) or Parental (panel **B**), was assessed upon treatment with OTX-008 alone (5 μM for SK-MEL-28 and 60 μM for A375), or with EG00229 alone (12.5 μM), or with a combination of the two drugs. Negative controls are represented by cells treated with vehicle alone (DMSO); Parental drug-sensitive cells were also analyzed in the presence of PLX-4720 (2 μM).

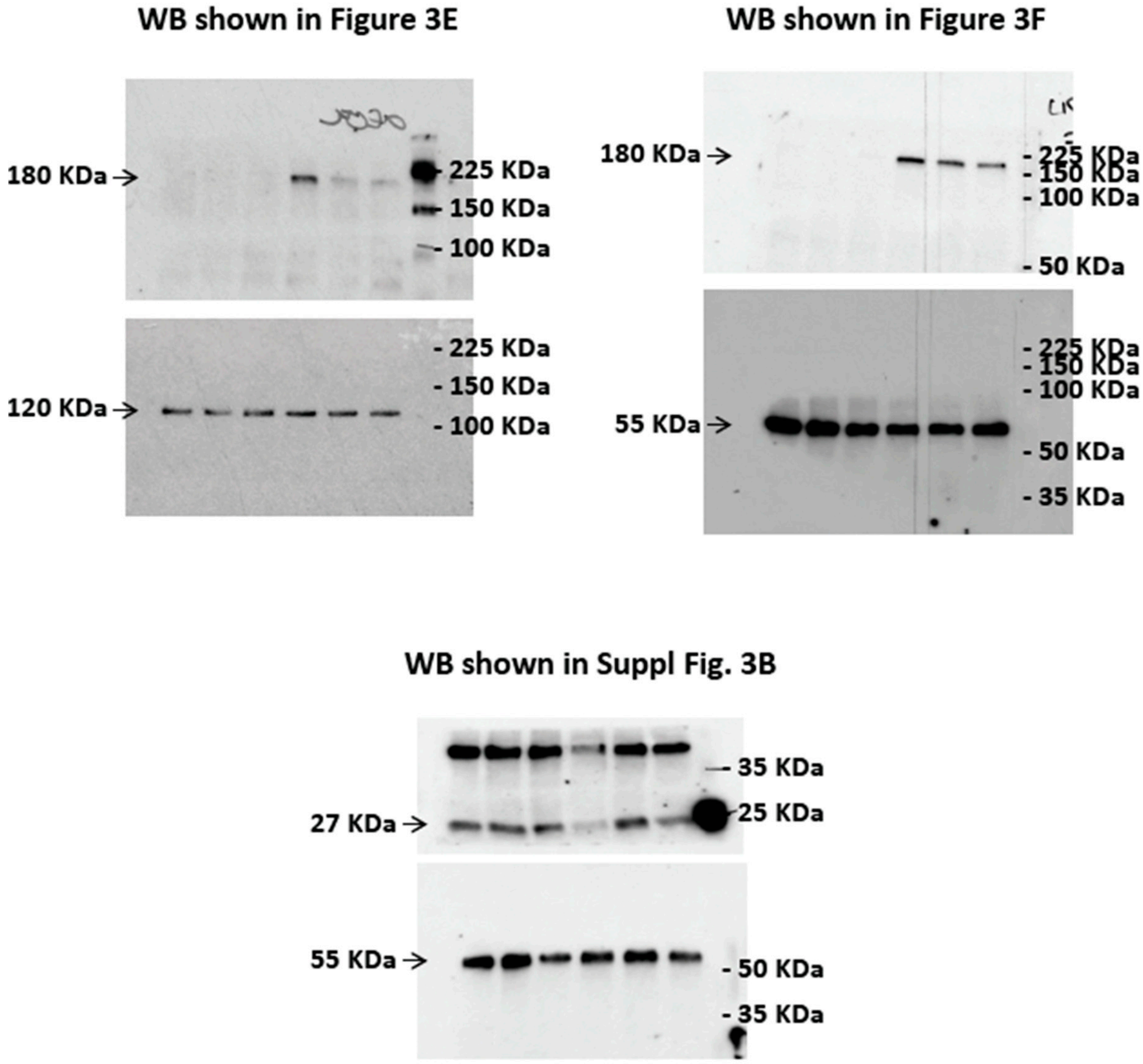

Figure S6. Uncropped immunoblots shown in Figures 3E-3F and Figure S3B.

© 2020 by the authors. Licensee MDPI, Basel, Switzerland. This article is an open access article distributed under the terms and conditions of the Creative Commons Attribution (CC BY) license (http://creativecommons.org/licenses/by/4.0/).
